# Whipple’s endocarditis and biologic response modifiers: a case-based review of an intricate host-pathogen interaction

**DOI:** 10.1007/s15010-026-02828-5

**Published:** 2026-06-03

**Authors:** Lorenzo Bertolino, Raffaella Gallo, Anna Maria Carolina Peluso, Fabian Patauner, Oriana Infante, Fabio Luciano, Nathalie Scarpulla, Marcella Sannino, Antonella Vulcano, Luigi Panico, Emanuele Durante-Mangoni

**Affiliations:** 1 Department of Precision Medicine, University of Campania ‘L. Vanvitelli’, Napoli, Italy; 2 Department of Advanced Medical & Surgical Sciences, University of Campania ‘L. Vanvitelli’, Napoli, Italy; 3 Unit of Gastroenterology and Digestive Endoscopy, AORN Ospedali dei Colli-Monaldi Hospital, Napoli, Italy; 4 Unit of Microbiology and Virology, INMI “L. Spallanzani”, Rome, Italy; 5 Unit of Anatomy and Pathological Histology, AORN Ospedali dei Colli-Monaldi Hospital, Napoli, Italy; 6 Unit of Internal Medicine and Transplants, AORN Ospedali dei Colli-Monaldi Hospital, Napoli, Italy

**Keywords:** Whipple's disease; Tropheryma whipplei; blood-culture negative infective endocarditis; biologic response modifiers; IL-17 inhibitors

## Abstract

Whipple’s disease is a rare and chronic multisystem infectious disorder caused by *Tropheryma whipplei*. Blood-culture negative infective endocarditis may complicate the course of classical Whipple’s disease or appear as an isolated manifestation. The pathophysiology of this rare and complex disorder is still unclear, and the appropriate therapeutic management is debated. Recent reports have highlighted the role of biologic response modifiers in causing overt disease or in accelerating patient presentation by worsening pre-existing symptoms especially when administered in patients with isolated joint manifestations. In this clinical scenario, we describe the first reported case of classical Whipple’s disease complicated with endocarditis in which patient symptoms were exacerbated by administration of IL-17 inhibitors. The patient presented with a history of long-standing fever and seronegative spondylarthritis and received a diagnosis of native aortic valve blood-culture negative infective endocarditis. Associated gastrointestinal symptoms prompted duodenal endoscopy with histology and molecular biology confirming the diagnosis. The following review provides valuable insights in the pathophysiology, diagnosis, treatment and long-term management of Whipple’s disease and underlies how modern medicine and newer treatment options are shaping the host-pathogen interaction and presentation patterns of infectious diseases.

## Introduction

*Tropheryma whipplei* is a Gram-positive actinobacterium firstly discovered in 1990s. The clinical spectrum of human disease may vary from isolated organ involvement to multisystem chronic disorder known as classical Whipple’s disease (CWD) [1]. Blood-culture negative infective endocarditis (BCNIE) is a rare manifestation of *T. whipplei* infection that occurs more commonly as an isolated manifestation rather than in the context of CWD. The pathophysiology of this infection relies upon a complicated host-pathogen interaction in which the immune system plays a major role.

Biologic response modifiers (BRMs), also known as immunomodulators, are a heterogeneous group of drugs with a major clinical application in autoimmune disorders and cancer immunotherapy. BRMs are also extensively used to treat rheumatological disorders such as rheumatoid arthritis, psoriatic arthritis and seronegative spondyloarthritis. These drugs act by controlling different steps of the immune response associated with exaggerated or abnormal immune system activation and unequivocally represent a milestone in the immunology of the past decade. By contrast, the disruption of immune homeostasis and the unbalanced stimulation and suppression of the immune system induced by BRMs is responsible of a wide range of adverse effects [2]. A specific risk of viral, bacterial, mycobacterial and/or fungal infectious disorders is associated with almost all BRMs and has especially been reported for TNF antagonists and anti-CD20. To a lesser extent, also IL-17 inhibitors (IL-17i), such as secukinumab, are associated with infectious complication and both fungal and viral super-imposed infection are reported [3, 4].

In the last 20 years, several authors have described a strict association between BRMs and WD symptoms onset or exacerbation [5–8]. Isolated joint manifestations are common at WD onset with highly heterogenous patterns of axial and peripheral joint involvement and can often be misdiagnosed with undifferentiated polyarthritis or seronegative spondyloarthritis [1]. Those patients can receive administration of BRMs that ultimately disrupt the host-pathogen interaction leading to WD symptoms exacerbation [9]. Due to the rapidly expanding number and indications of BRMs, patients with unrecognized WD-related isolated joint disease that will receive BRMs will likely increase.

In this article, we report the first clinical case of CWD complicated with endocarditis in which patient symptoms were exacerbated by administration of IL-17i and offer a case-based review of the most important diagnostic and therapeutic challenges of WD and how BRMs can shape the host-pathogen interaction and presentation patterns of infectious diseases.

### Case report

A 43-year-old Caucasian man was evaluated for a three-year history of intermittent fever and joint pain. He was a lawyer and an active smoker (3 pack-years). He denied use of illicit drugs and had no relevant travel history. His past medical history was significant for seronegative spondylarthritis - diagnosed two years before current presentation, treated with IL-17i (secukinumab) and low dose prednisone (5 mg qd) - and for glucose-6-phosphate dehydrogenase deficiency (G6PDD) with symptomatic haemolytic crises during childhood. On clinical history taking, fever had indeed always been responsive to repeated courses of amoxicillin-clavulanate, with relapse after discontinuation. Fever was initially low-grade but worsened 1-year before admission, peaking at 39 °C, shortly after the first three administrations of secukinumab (300 mg subcutaneously every week). Five months before admission, he developed abdominal pain, diarrhoea and weight loss (8 kg, 10% of body weight). At admission the patient was in no acute distress but appeared chronically ill, febrile (body temperature 38.3 °C), with normal vital parameters. On physical examination he appeared well oriented to place and time but with slightly slowed mentation and spontaneous bilateral horizontal nystagmus. An early diastolic Erb’s area murmur was detected at cardiac auscultation. The remainder of the clinical examination was unremarkable. Blood tests showed marked leucocytosis with neutrophilia, normocytic anaemia, increased C-reactive protein (9.5 mg/dL) and erythrocyte sedimentation rate (68 mm/h) and features of malnutrition (low levels of triglycerides, total cholesterol and high-density lipoprotein cholesterol, multiple vitamin deficiencies and hypoalbuminemia) (Table 1).

**Table 1:** Baseline blood test results on admission

	Result	Reference range
White blood cells, cells/mcL	21,000	4500–11,000
Neutrophils, cells/mcL	18,000	2000–5000
Hemoglobin, g/dL	10	13–18
Mean corpuscular volume, fL	80	76–96
C-reactive protein, mg/dL	9.5	< 0.5
Erythrocyte sedimentation rate, mm/1-h	68	2–14
Creatinine, mg/dL	1.3	0.7–1.1
Lactate dehydrogenase, UI/mL	266	100–246
Beta2-microglobulin, mg/dL	3.2	1.4–2.2
Rheumatoid factor	< 10	< 10
Triglycerides, mg/dL	49	50–150
Total cholesterol, mg/dL	64	< 200
Albumin, g/dL	2.6	3.5–5
International normalized ratio	1.44	0.8–1.2
Fecal calprotectin, mcg/g	87	< 50
Fecal occult blood test	Positive (++)	Negative

**Fig. 1:**
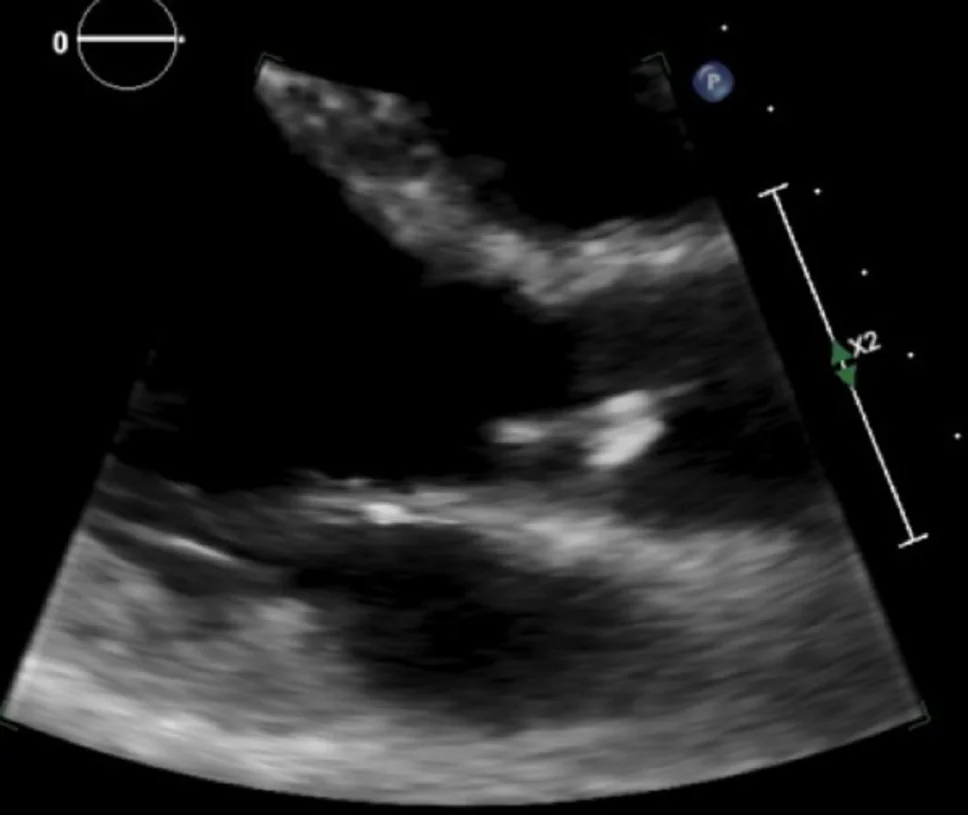
Transthoracic echocardiography with a parasternal long axis view showing a vegetation (14 mm hyperechogenic mobile mass) on the non-coronary cusp of the native aortic valve

As part of a standardized protocol in patients with fever and a cardiac murmur, a transthoracic echocardiography (TTE) was performed, showing mild aortic valve regurgitation with a vegetation (14 × 7 mm) on the non-coronary cusp (Fig. 1) and a second smaller vegetation on the right coronary cusp. Six sets of blood cultures, collected off any recent antimicrobial administration, were all negative. Serology for *Coxiella burnetii*, Bartonella spp. and Brucella spp. were negative together with screening for syphilis and HIV. Stool test for parasites and urine culture were negative. Antinuclear and anti-phospholipid antibodies and rheumatoid factor were undetectable. Considering the subacute blood culture negative infective endocarditis (BCNIE) and fever responsiveness to antibiotics, empirical antibiotic treatment was started with intravenous (IV) amoxicillin-clavulanate (2.2 g qid) and gentamicin (3 mg/kg qd) for 2 weeks, resulting in prompt fever resolution and a sharp decrease of inflammatory markers.

**Fig. 2:**
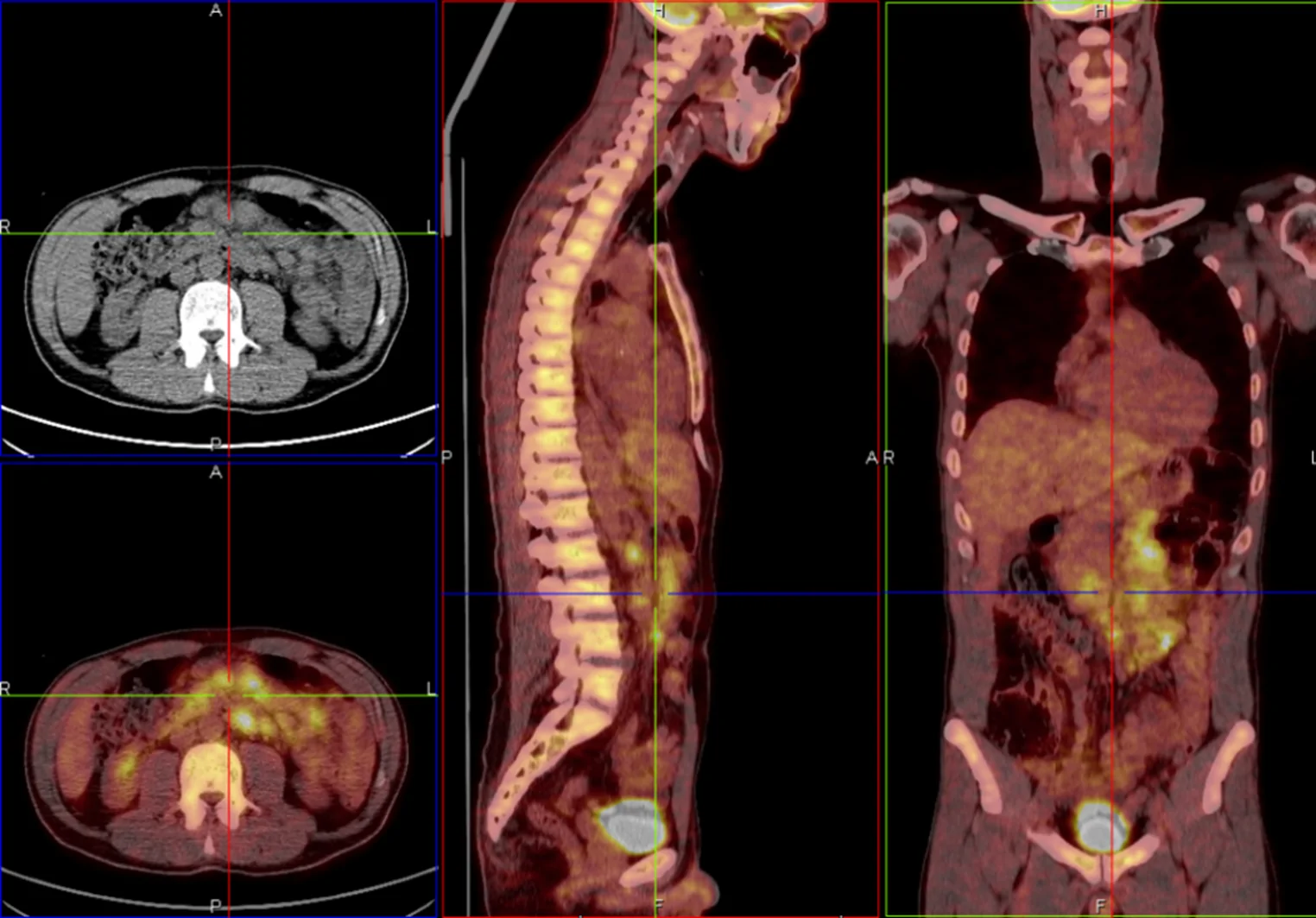
Positron emission tomography with 2-deoxy-2-[fluorine-18] fluoro-D-glucose integrated with computed tomography (18 F-FDG PET/CT) performed at diagnosis showing marked tracer uptake (SUVmax 10.2) of celiac, mesenteric, peripancreatic, para-aortic, intercaval-aortic and greater omental lymphadenopathies

**Fig. 3:**
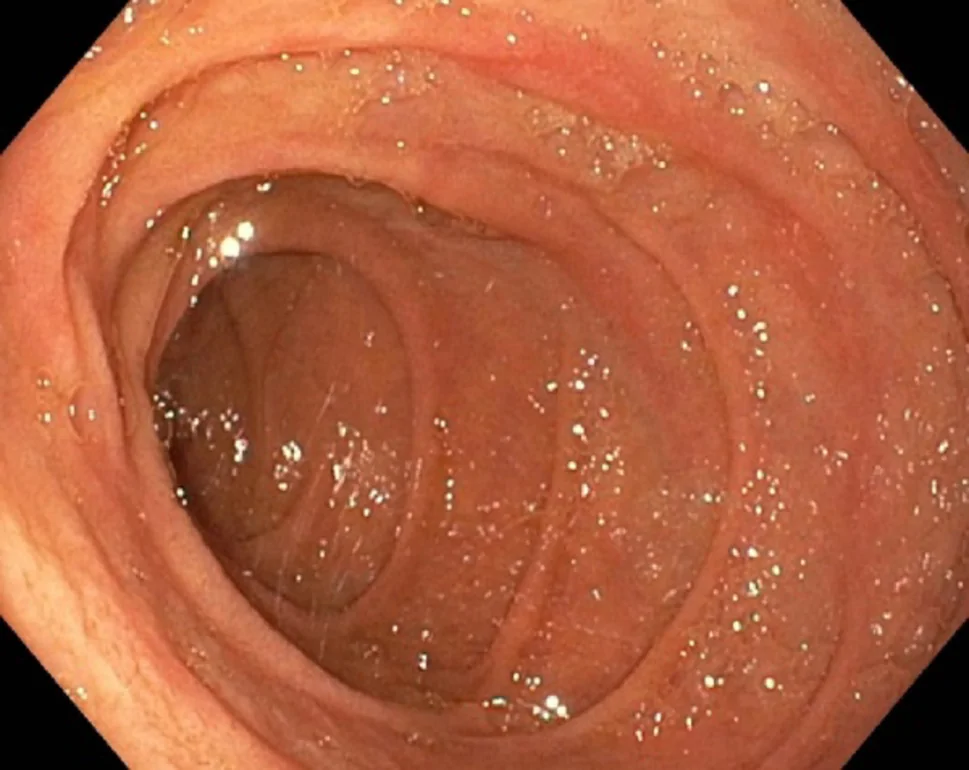
Upper gastrointestinal endoscopy performed at diagnosis showing normally appearing duodenum

**Fig. 4:**
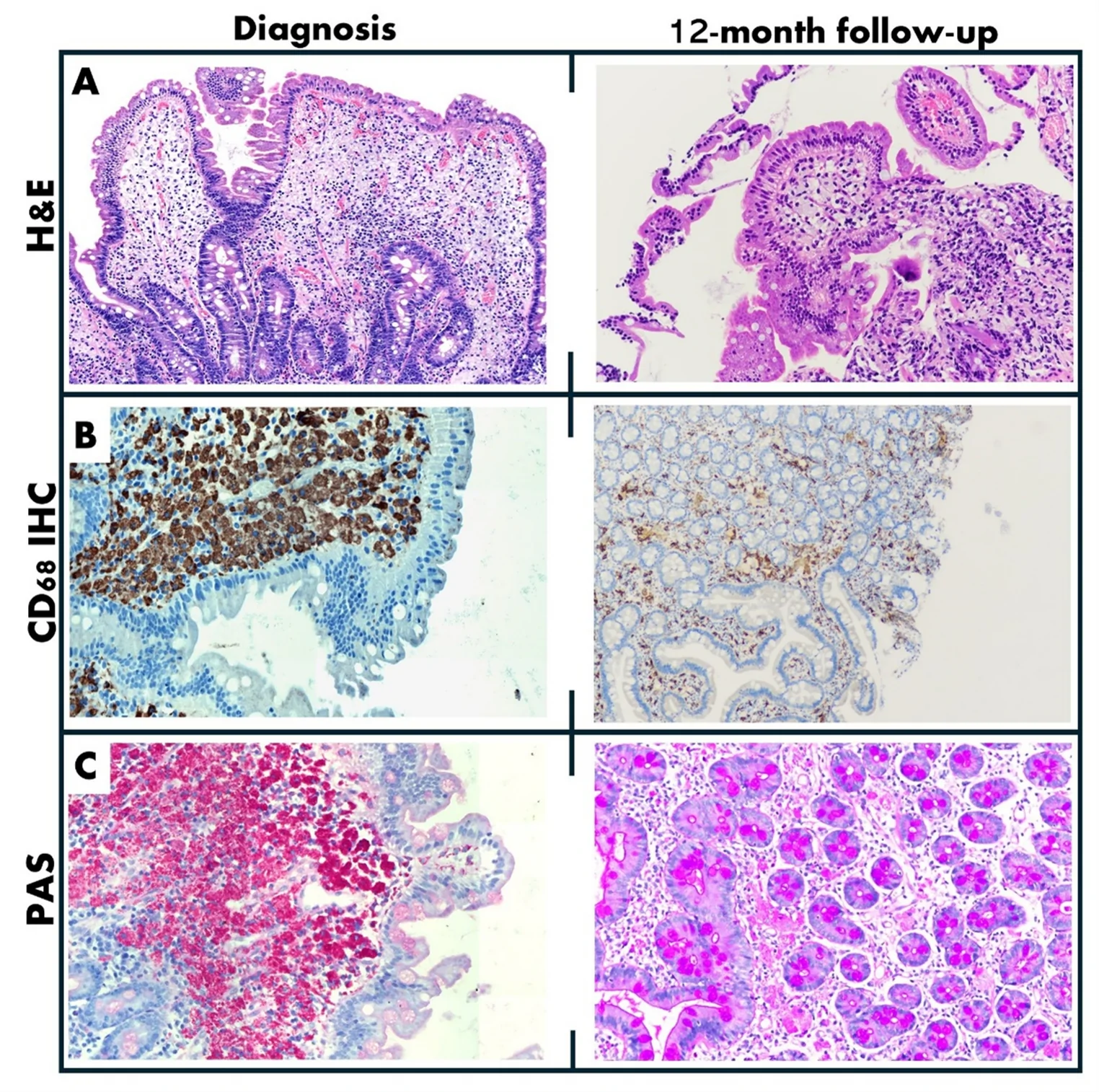
Duodenal mucosal histological findings at diagnosis (on the left) and 12-month follow-up (on the right. Panel A: hematoxylin and eosin (H&E) staining showing intense chronic inflammatory infiltrate in the lamina propria. Panel B: Immunohistochemistry (IHC) confirming the infiltrate was mostly made up of foamy macrophages (CD68PGM1+). Panel C: Periodic acid-Schiff (PAS) staining demonstrating macrophage cytoplasm containing large amounts of PAS-positive particles

Abdominal ultrasound (US) showed celiac, mesenteric, peripancreatic, para-aortic, intercaval-aortic and greater omental lymphadenopathies, confirmed by ^18^FDG-PET/CT scan (SUV max 10.2) (Fig. 2). Upper gastrointestinal (GI) endoscopy was performed to investigate possible malabsorption. Biopsy specimens of a normally appearing duodenum (Fig. 3) were obtained, and histological examination showed a diffuse and intense inflammatory mucosal infiltrate consisting of foamy macrophages (CD68+) containing intracytoplasmic granules that were positive on periodic acid Schiff (PAS) staining (Fig. 4).

The clinical scenario of BCNIE coupled with such histological findings on bowel biopsies raised suspicion of Whipple’s disease (WD). Brain MRI and lumbar puncture were performed to investigate central nervous system (CNS) involvement. The MRI was negative and cerebrospinal fluid examination was unremarkable. PCR for *Tropheryma whipplei* was requested on blood, duodenal biopsies and cerebrospinal fluid. PCR on small bowel biopsies (paraffin sections) resulted positive, confirming the diagnosis of classic WD (CWD) with cardiac involvement.

Once the etiological diagnosis of BCNIE was reached, therapy was switched to IV ceftriaxone 2 g qd for 2 weeks. Because of the absolute contraindication to trimethoprim/sulfamethoxazole (TMP/SMX) administration (severe G6PDD, confirmed on repeat testing), the patient was discharged on oral therapy with doxycycline 100 mg bid plus hydroxychloroquine (HCQ) 200 mg bid, planned for at least 12 months. Of note, the patient had already been treated with HCQ for seronegative spondylarthritis before starting IL17i, in the absence of adverse haemolytic reactions.

At 6 months follow-up, the patient was afebrile with normalised inflammatory markers. Diarrhoea and arthralgia had resolved, and body weight increased by 3 kg. TTE was negative for any remnant vegetation and PET/CT scan showed reduced metabolic uptake of abdominal lymph nodes (SUV max 2.76).

At 1 year follow-up, the patient underwent follow-up GI endoscopy with duodenal biopsy showing persistence of the chronic inflammatory infiltrates with PAS positive macrophages, mildly reduced when compared with the baseline sample (Fig. 4). Repeat PCR for *T. whipplei* on duodenal tissue was negative. Antimicrobial therapy was well tolerated and maintained up to 18 months. At 5-year follow-up, the patient remains in good clinical conditions, without clinical and/or biochemical evidence of relapse.

## Discussion

WD is a rare and chronic multisystem infectious disease usually affecting middle-aged white men [1, 10]. The rarity of this disorder seems to be more likely associated to a complex host-pathogen interaction and unspecific clinical manifestations rather than the real spread of the causative agent, which is considered universally distributed. The changing landscape of modern medicine and the increasing rate of comorbid patients with a reduced net state of immunocompetence may potentially increase the number of cases in which *T. whipplei* becomes able to escape the immune system, causing overt disease. Our clinical experience offers an insight into the pathophysiology and diagnostic-therapeutic management of this under-recognized disorder and provides an example of how modern treatment strategies are shaping the host-pathogen interaction, modifying epidemiology and presentation patterns of infectious diseases.

### Bacteriology and epidemiology

*T. whipplei*, the causative agent of WD, is a Gram-positive rod-shaped intracellular actinobacterium [11]. Its genome, first described partially by Wilson et al. in 1991, contains only few genes pertaining to pathogen metabolism making it strictly depending on the infected host cells for both enzymes and substrates [12, 13]. By contrast, up to 70% of *T. whipplei* genome encodes surface proteins that carry a great degree of sequence variation, underlying the complex interaction of this microorganism with the host immune system [14]. It has been postulated that this microorganism exists in two biological forms: one dormant extracellular form where the bacteria lay in large aggregates and one replicating form dwelling into vacuoles of infected eukaryotic cells [15]. *T. whipplei* is ubiquitous in the environment and shows a faecal-oral route of transmission [15]. Asymptomatic GI carriage identified using stool PCR testing has been detected among humans with a prevalence of ≈ 4% [16]. This prevalence increased at 12% in workers of sewage management facilities, suggesting that exposure to this agent may be increased in some categories [16]. Other authors found a higher prevalence of *T. whipplei* GI carriers in northern Spain (around 9%), peaking at 33% in people living with HIV [17]. A slightly lower overall prevalence was found in northern Italy (4.9%). In that setting, younger age, male sex and enteric protozoal coinfection were possible risk factors [18]. Male sex has historically been linked to an increased risk of WD but recent studies on large case series have found no significant differences between males and females [10, 15]. Despite the ubiquitous environmental distribution and the significant prevalence of *T. whipplei* carriers, only a minority of those infected by this agent will develop WD (< 0.01%) [16]. The exact WD incidence and prevalence are extremely difficult to predict. Recent data from northwestern Italy suggests a local WD prevalence of 3 cases per 1 million individuals but other studies suggest that this may be higher in other geographical areas such as the United States where WD prevalence is estimated to be 9.8 cases per 1 million individuals [10, 19].

### Pathophysiology and association with biologic response modifiers

Development of clinically overt WD strictly depends on a complex host-pathogen interaction between the host immune system and the microorganism which is intimately linked to genetic, environmental and pathogen factors [15]. A predisposing genetic background may partially explain the altered peripheral and mucosal T cell response towards *T. whipplei* as suggested by the WD association with certain HLA haplotypes, such as HLA-B27 [20–22]. More recently, HLA-DRB1*13 and DQB1*06 alleles were found to be significantly associated to WD [23]. Moreover, T-cell induced cytokine production has a well-recognized genotypic profile in WD and patients developing WD have a significantly deviated rate of TGF-β functional phenotype with a greater frequency of TGF-β low producer [24]. This genetic background favours a suppressed Th1 response and a shift towards functional Th2 activation, promoting clinically overt WD [20–25]. Once internalized in host cells, the bacterium tackles the inflammatory response by impairing antigen presentation and maturation of macrophage phagosomes [26]. Together with an enhanced Th2 response, these patients also show a reduced Th17 activation. In the study by Biagi and colleagues, a low Th17 cytokine producer phenotype was strongly associated with WD [24]. Th17 are specialized T lymphocytes playing a pivotal role in mucosal immune response to several infections [27–29]. This T helper lineage acts by secreting a specific subset of cytokines, including IL-17 A, IL-17 F, IL-22 and IL-26, responsible for chemokines and antimicrobial protein production [30]. In addition, IL-17 can also be produced by NK and other T cell subtypes increasing the complexity of mucosal response to the infection [31]. These data suggest that IL-17 is of utmost importance in orchestrating the host immune response to *T. whipplei* and, therefore, IL-17i may disrupt this complex interplay and increase the risk of developing overt WD in asymptomatic carriers or exacerbate symptoms in patients with already established disease. To date, robust evidence regarding this association is lacking. In our case, we observed a clear temporal link between IL-17i administration and worsening of pre-existing WD symptoms. Further studies on larger case series are needed to confirm our hypothesis.

More compelling data are available regarding the association between WD and other biologic response modifiers (BRMs), such as tumour necrosis factor inhibitors (TNFi). An interesting case-control study found that administration of TNFi in the setting of undetermined arthropathy or seronegative spondylarthritis can worsen diarrhoea or fever and lead to overt WD [5]. In this study, patients with WD diagnosis after TNFi administration were compared with patients with WD who did not receive prior TNFi; of note, the incidence of WD complications and endocarditis was significantly higher in the former group (12.2% vs. 1.6%; *p* < 0.05) [5]. A link between TNFi administration and WD onset has been reported also by other authors [6–8]. Other BRMs have been linked to fever worsening or onset of GI manifestations in patients subsequently diagnosed and treated for WD, including anti-IL6 (tocilizumab) and IL-1 receptor antagonists (anakinra) [9].

A common clinical pattern shown by these patients is the preexisting elusive WD symptoms, mostly represented by joint manifestations that are followed by the onset of GI symptoms several months after starting immunomodulatory drugs.

### Diagnosis

Diagnosis of WD can be extremely challenging and requires a two-step approach with recognition of typical histological findings and confirmation through direct isolation or molecular detection of *T. whipplei* in the involved organ [31]. Being *T. whipplei* a slow-growing microorganism, with a replication time of about 18 days, culture is challenging, takes several months and requires specialised techniques. Therefore, isolation of *T. whipplei* relies on molecular biology techniques able to detect specific microbial DNA sequences. *T. whipplei*-specific quantitative polymerase chain reaction (qPCR) is a reliable method for diagnosing WD and should be confirmed by sequencing several pathogen target genes to avoid false-positive results. Indeed, qPCR results must be interpreted with caution as positive results may occur in healthy carriers or in the presence of other phylogenetically related bacteria. In addition, false-negative results may also be obtained on paraffin section testing [32]. Our case highlights that the selection of the appropriate tissue sample for *T. whipplei* PCR testing is of utmost importance. Indeed, PCR was positive only on duodenal tissue and negative on peripheral blood and CSF, despite of BCNIE and definite CNS symptoms.

In CWD with associated malabsorption syndrome, the duodenum can be macroscopically involved showing swollen and grey-yellowish mucosa, with flattened folds and diffuse patchy whitish areas [33, 34]. However, these changes are uncommon and duodenal histological examination should be performed also in patients with normal appearing mucosa [32].

Fluorescence in-situ hybridization (FISH) can be used to detect *T. whipplei* within tissue samples by using fluorescently labelled DNA probes. FISH can be applied on cytological smears of fluids and formalin-fixed, paraffin-embedded (FFPE) tissue. When FISH is combined with immunofluorescence labelling, it can detect *T. whipplei* both within and outside macrophages, improving diagnostic yields. FISH is influenced by previous treatments and persistently positive results may also indicate persistence or recurrence [35].

Metagenomic next-generation sequencing (mNGS) may represent a valuable tool for WD diagnosis, allowing pathogen detection in different samples. *T. whipplei* detection trough mNGS in bronchoalveolar lavage of patients with acute pneumonia has been reported but a direct pathogenic link of this finding still needs to be established [36]. In addition, mNGS allows detection of drug-resistance genes in *T. whipplei* genome, highlighting a possible role of this technique in guiding WD treatment [37]. mNGS is an unbiased diagnostic tool and possibly amplifies all nucleic acids in a sample. Therefore, it may represent a useful tool in unbiased approaches. However, it is crucial to combine results of mNGS with clinical pre-test probability of the disease and histological findings of involved organs to reduce false-positivity or avoid unnecessary long-term treatment courses in asymptomatic carriers.

### **Clinical** presentation

**Fig. 5:**
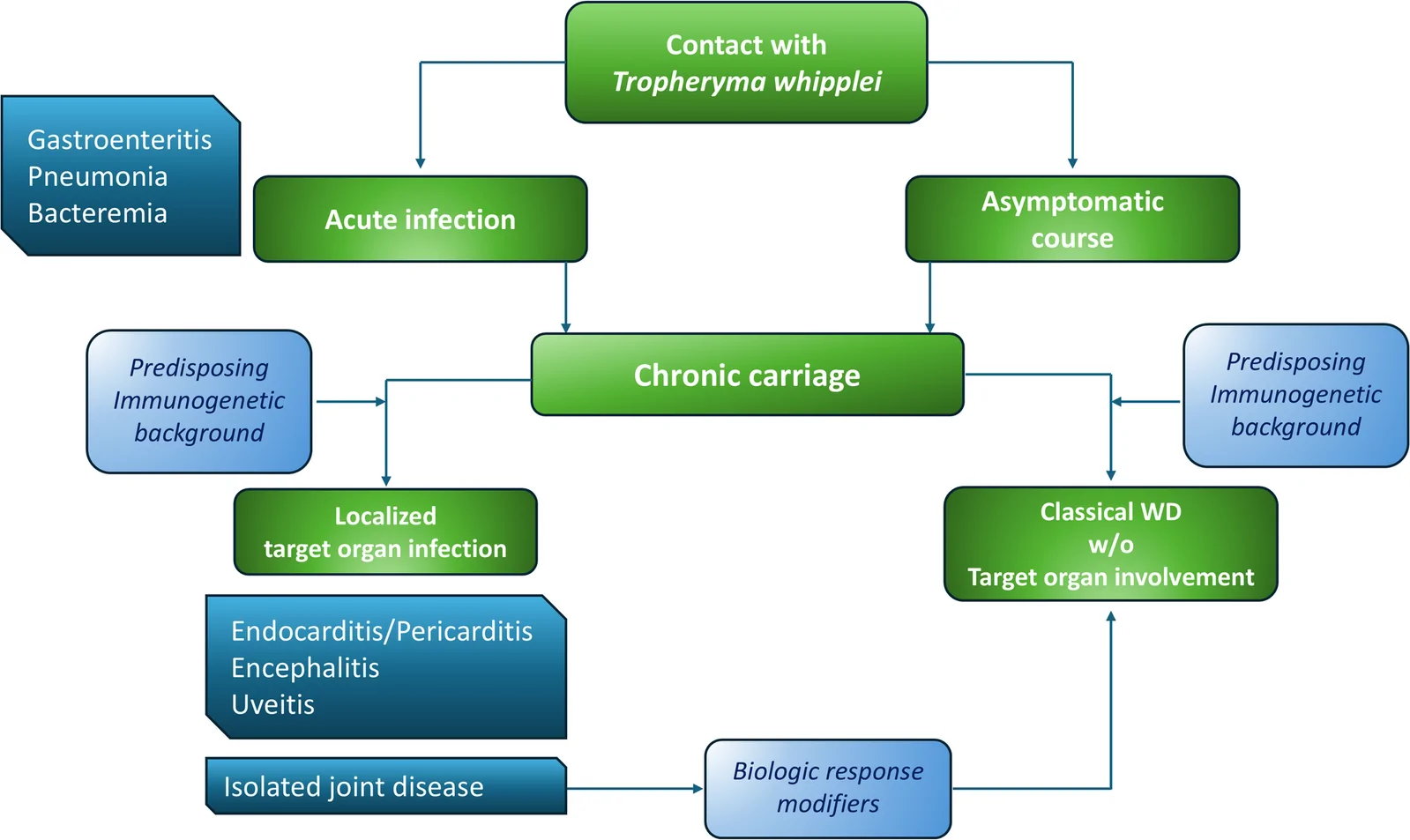
Flowchart showing the spectrum of Whipple’s disease from initial contact with the culprit microorganism to acute and chronic infections. The showed pathophysiological model highlights the impact of a predisposing immunogenetic background in favouring the transition from chronic carriage to localized target organ infection or Classical Whipple’s disease. Biologic response modifiers can increase the risk of developing Classical WD (with or without target organ involvement) in patients with isolated joint disease

The clinical presentation of WD can be extremely diverse, including localized or systemic involvement with acute or chronic onset. Contact with *T. whipplei* can be followed by acute infections (pneumonia, gastroenteritis, bacteremia) or an asymptomatic course. Both conditions may evolve into a chronic carriage state and, in the presence of a favourable immunogenetic background, develop into symptomatic stages with chronic localized infections (cardiac, CNS, osteoarticular) or CWD (Fig. 5). The latter is a chronic multisystem disease characterized by chronic diarrhoea, weight loss and joint pain, occurring in 76%, 92% and 67% of cases, respectively [1, 16]. A certain degree of joint involvement is almost universal in CWD and can be extremely heterogeneous, involving peripheral joints (oligo-polyarthritis) or vertebral spine (spondylarthritis) [32]. In more recent studies, the rate of joint involvement was above 90% in CWD [38]. Rheumatological manifestations of WD can be misleading. As occurred in our case, a majority of WD patients with isolated joint manifestations seeks specialist rheumatological assessment for undetermined arthropathy and receive a diagnosis of inflammatory rheumatic disease, such as seronegative spondylarthritis, that often fails to respond to disease-modifying antirheumatic drugs, prompting administration of BRMs. Gastrointestinal involvement is also part of the cardinal manifestations of CWD. A malabsorption syndrome is found in more than 90% of patients at diagnosis [38]. Lymphadenopathies and abdominal pain are commonly seen in around 50–60% of patients with WD [32]. Weight loss is usually preceding diagnosis by around 6 months, whereas the time from first symptoms (usually osteoarticular pain) to CWD diagnosis is 5–8 years [1, 38]. This suggests that onset of malabsorption syndrome and weight loss in a patient with prior (long-standing) history of joint pain should prompt suspicion of WD and often aids the Clinician to reach the final diagnosis.

Cardiac involvement in WD is isolated in most cases but can occur also in the context of CWD [32]. Postmortem studies have reported PAS-positive macrophages in endocardium, myocardium and pericardium of almost 80% of cases [39]. However, clinically overt cardiac involvement in CWD is uncommon and may occur as pericarditis or BCNIE representing a diagnostic and therapeutic challenge. BCNIE is commonly associated with prior antimicrobial administration, but in selected patients it should prompt investigation for atypical or fastidious microorganisms [40, 41]. This includes guideline-recommended serology testing for *Coxiella burnetii*, *Bartonella* spp., *Aspergillus* spp., *Mycoplasma pneumoniae*, *Brucella* spp. and *Legionella pneumophila* and/or molecular biology on blood and tissue for *Tropheryma whipplei*, as well as newer techniques such as broad range 16 S ribosomal RNA amplification and cell-free DNA detection [41, 42]. In the study by Geißdörfer W et al., *T. whipplei* was detected trough bacterial 16 S rRNA amplification and confirmed by specific PCR in the valve tissues of 16 out of the 255 patients with bacterium-positive cardiac valves (6.3%) [43]. Other studies suggest that *T. whipplei* accounts for 2.6%-6.3% of all BCNIE [44].

Prior administration of BRMs, such as TNFi, increases the risk of cardiac involvement in this setting [5]. *T. whipplei* BCNIE is usually slow progressing, with a subacute or chronic clinical presentation. The median time between symptom onset and diagnosis is 5 [0.16-20] years. The aortic valve is the most common location and vegetations are found in approximately 70% of cases [16, 45]. At histology, affected valves are characterized by prominent fibrosis and low-grade inflammation [46].

CNS involvement is a worrisome complication and has been found in 6 to 60% of patients with CWD [32]. PCR of CSF is positive for *T. whipplei* DNA in almost 50% of them [38]. Cognitive dysfunction is among the most common neurological signs and includes dementia, memory loss and confusion. Personality changes and depression are observed in about 50% of patients with CNS-WD. Nystagmus is also seen in this setting (14%) and can be accompanied by synchronized contractions of the masticatory muscles, namely oculomasticatory myorhythmia (OMM), a pathognomonic although rare (2%) manifestation of WD [38, 47]. Despite the absence of OMM, our patient showed nystagmus and mild cognitive impairment that prompted suspicion of neuro-Whipple. However, we were unable to detect overt signs of CNS involvement at brain MRI and CSF examination.

### Treatment and long-term follow-up

Treatment of WD with cardiac involvement is based on expert opinion and low-quality evidence, being this disorder extremely rare. Doxycycline and SMX have shown to be active in vitro against *T. whipplei* [48]. Of note, TMP alone is not effective, since *T. whipplei* lacks dihydrofolate reductase [49–51]. Doxycycline alone is bacteriostatic but the combination with HCQ has synergistic properties, making doxycycline bactericidal [32]. Current guidelines recommend a prolonged (≥ 18 months) course of doxycycline (200 mg/day) plus HCQ (200–600 mg/day) [42]. A recent trial conducted in patients with CWD showed non-inferiority of oral treatment with doxycycline and HCQ when compared to an initial 2-week intravenous course of ceftriaxone followed by oral TMP-SMX for 12 months [38]. However, the study included 60 patients with CWD and only a minority (15%) showed cardiac involvement, that was classified as both endocardial and/or pericardial. Moreover, patients with lone *T. whipplei* endocarditis were excluded. In the Spanish cohort of 17 patients with BCNIE caused by *T. whipplei*, a majority received an initial course of intravenous ceftriaxone (64.7%) for at least 2 weeks, followed by TMP/SMX 160/800 mg bid for at least 1 year in 70% of cases and doxycycline 200 mg/day plus HCQ 600 mg/day in the remaining 30%. Outcome was satisfactory in all patients after a median follow-up of around 28 months [45]. Therefore, it seems reasonable to still consider an initial intravenous antimicrobial course in those who present with BCNIE.

In our case, SXT administration was hampered by concomitant severe G6PDD. HCQ has historically been linked to haemolytic reactions in such patients, but can now considered at low risk [51].

After an initial intravenous course of ceftriaxone 2 gr/day for 2 weeks, we decided to switch our patient to oral treatment with doxycycline 200 mg/day plus HCQ 400 mg/day, without observing adverse events.

Immune reconstitution inflammatory syndrome (IRIS) occurs in up to 20% of patients with CWD usually in the first two weeks after starting treatment. It is important to underline that patients receiving immunosuppressant medications have been linked to an increased risk of IRIS (up to 90%) [52].

Optimal duration of treatment in Whipple’s endocarditis is unknown, and antimicrobial administration should be considered for at least 18 months according to current recommendations [42]. Histological changes may persist years after successful treatment. In the past, repeat PCR on involved tissues was proposed to monitor response to therapy, but evidence of its usefulness is still scarce [53]. Interpretation of follow-up histological and PCR findings in patients with WD is a controversial issue and should be done with a case-by-case approach and in referral centres. A prolonged treatment course (beyond 18 months) could be considered in patients without clear clinical and haematochemical response and showing persistently positive histology and PCR. Our experience confirms that persistent histological changes are not unequivocally associated with infection persistence and suggests that in patients with good clinical/biochemical response and a negative follow-up PCR, treatment could be discontinued after a full 18-month course. However, risk of relapse remains difficult to predict, and recent cohort studies have suggested that may be as high as 50% after a median follow-up of 27 months. Risk factors for relapse in this cohort were older age, male sex and delayed diagnosis [54]. Therefore, independent of the chosen therapeutic strategy, a structured follow-up is warranted in all patients treated for WD.

## Conclusions

WD remains an extremely rare disorder but given the non-specific clinical presentation and the diagnostic challenges it might be overlooked more often than supposed. Management issues dramatically prolong the time between symptom onset and diagnosis, increasing the risk of neurological or cardiac complications. When left untread, WD is almost universally fatal, but when recognized and appropriately treated patients may show a favourable outcome. Our clinical case recapitulated most of the diagnostic and therapeutic challenges of WD, and specifically in the subgroup of patients with endocardial involvement. Moreover, this clinical case offers insights into the pathophysiology of this disorder, highlighting the need for an appropriate and strict selection of candidates to immunomodulatory agents among patients with rheumatological manifestations, in whom unexplained symptoms, such as long-standing fever or worsening osteoarticular involvement, may prompt consideration of this underlying infection.

It is crucial to foster discussion on the appropriate management of this rare disorder, that is still based on low quality evidence, to improve patient outcomes and reduce risk of long-term complications and relapse.

## Data Availability

No datasets were generated or analysed during the current study.
